# Evaluation of Situational Judgment Tests in student selection in Indonesia and the impact on diversity issues

**DOI:** 10.1186/s12909-022-03247-4

**Published:** 2022-04-02

**Authors:** Diantha Soemantri, Ardi Findyartini, Sophie Yolanda, Emma Morley, Fiona Patterson

**Affiliations:** 1 Department of Medical Education, Faculty of Medicine, Universitas Indonesia, Jakarta, Indonesia; 2 Medical Education Center, Indonesia Medical Education & Research Institute (IMERI), Faculty of Medicine, Universitas Indonesia, Jakarta, Indonesia; 3 Department of Physiology, Faculty of Medicine, Universitas Indonesia, Jakarta, Indonesia; 4 Work Psychology Group, Derby, UK; 5 School of Medicine, University of Nottingham, Nottingham, UK

**Keywords:** Situational judgment test (SJT), Undergraduate, Medical students, Selection, Non-academic attributes

## Abstract

**Background:**

Internationally, medical selection relies heavily on prior academic attainment which has an adverse impact on the diversity of selected students. Since non-academic attributes are also important, this study aims to evaluate the use of a Situational Judgment Test (SJT) for selection and the impact on student diversity relating to gender, ethnicity and socio-economic status. Previous SJT research has almost entirely originated from a Western context and this study focuses on new evidence in a South East Asian context with a different demographic profile.

**Methods:**

Thirty faculty members developed 112 SJT scenarios assessing professionalism, communication and self-awareness domains. The scenarios underwent a concordance stage where stakeholder input was sought on the content appropriateness, to define the item scoring key, followed by an initial psychometric evaluation with first and second year medical students (*N* = 436). Based on these results, 30 scenarios, consisting of 128 nested items, were selected for pilot testing and evaluation regarding diversity issues with two cohorts of applicants in 2017 (*N* = 446) and 2018 (*N* = 508).

**Results:**

The SJT demonstrated good internal consistency (Cronbach’s alpha of 0.80 and 0.81 respectively). There were significant differences in SJT scores based on gender in both years, where females consistently outperformed males (*p* = .0001). However, no significant differences were found based on high school origin, parental educational background or ethnicity.

**Conclusions:**

This is the first study to evaluate the use of an SJT in Indonesia, which has a unique diversity profile compared to Western countries. Largely, the preliminary results replicate previous studies of the potential diversity benefits of using an SJT as a tool for medical student selection and has the potential to level the playing field regarding socio-economic status and ethnicity. Further studies exploring more variables representing diversity are warranted to confirm the early results in this study.

## Background

Historically, medical selection across the globe has focused heavily on indicators of academic attainment (such as academic records and science-based admission tests) to make selection decisions [1]. Although academic indicators are clearly important in medical selection, research consistently demonstrates this to have a negative impact on the diversity of students selected, especially for those students from more educationally disadvantaged backgrounds [2]. More recently, there is a growing recognition that a range of non-academic attributes are important and researchers have explored the use of different selection methods to assess these reliably [1].

Large scale systematic reviews [3] and a recent meta-analysis [4] provide good evidence of the reliability and validity of situational judgement tests (SJTs) to evaluate a range of personal attributes important for anyone entering a career in healthcare. Some studies have also shown SJTs to offer diversity benefits, especially relating to socio-economic status [5, 6]. However, it is important to note that this body of research presents data that arises almost entirely from a Western context. In a recent Ottawa consensus statement on selection and recruitment, researchers highlight that although the field of selection has developed considerably over the past 20 years, “*the majority of research and available material continues to originate from a limited number of global regions. Research and case studies from Asia, South America, the Middle East and Africa are lacking*” ([1], p. 8). It is important to note that health professional education generally, and selection and diversity issues specifically, cannot be isolated from the cultural and social structural context in which it takes place [7, 8]. There is also evidence that SJTs in particular may be culturally saturated [9], such that studies in other continents could offer new insights in the literature. For example, cultural differences between Western and Asian contexts in relation to professionalism, values [10] and communication style [11], may lead to different results regarding SJT validity, reliability and diversity benefits. This study presents the development, piloting and evaluation of a SJT for medical school admissions in a South East Asian context with a specific analysis relating to diversity issues, namely gender, ethnicity and socio-economic status. Examining the development and piloting of a locally designed SJT in this different context would also inform the design of future selection methods and policy in this region.

## Diversity issues and SJTs

### Gender

Consistent with independent studies where female candidates outperform male candidates in medical school admissions [12, 13], in their recent meta-analytic study of SJTs in healthcare, Webster et al. [4] demonstrate that females tend to marginally outperform males on SJTs. In terms of applying this to selection policy, it is important to consider how various selection methods are combined and/or weighted to address fairness. For example, most medical schools in the UK and in Australia use the UCAT (University Clinical Aptitude Test) for early screening in admissions. UCAT comprises both an SJT and four cognitive ability tests [5, 14]. In general, results show that males tend to outperform females on the cognitive ability tests and females outperform males in the SJT; which to some extent ‘levels the field’ regarding gender differences as part of medical school admissions [5].

### Socio-economic status

There is emerging evidence that SJTs may facilitate the diversification of enrolment into undergraduate and postgraduate medical education regarding socio-economic status. For example, Lievens et al. [5] demonstrated that an SJT used for medical school selection in the UK (part of the UCAT) does not favour applicants from higher socio-economic backgrounds; those from more educationally deprived backgrounds performed equally well on the SJT, when compared to those from independent fee-paying schools. Researchers have suggested that the negative effect of the cognitive tests on widening participation may be due to differential access to educational resources, for example access to coaching on certain selection methods, especially for candidates from educationally disadvantaged backgrounds [4]. Similarly, for postgraduate selection, Gardner et al. [15] found that an SJT for entry into surgical residencies provides a higher chance for underrepresented minorities to enter the programme. This was because those from underrepresented minorities performed better on an SJT when compared to a cognitive test, and so when the SJT was used to define progression to the next stage of selection, more of those from underrepresented minorities progressed to the next stage of selection [6].

### Ethnicity

In their UK study, Lievens et al. [5] identified significant differences between ethnic minority groups on an SJT compared to UK-white applicants, where those from ethnic minority groups tended to perform less well. Further studies need to be conducted to examine the underlying cause of these differences. Some researchers argue that SJT items are usually developed according to the cultural values of the ethnic majority group [16] and others also note that there is usually some cognitive element within SJT items, which may favour the majority group as shown in the study from Roth et al. [17]. When exploring overall selection policy, Patterson et al. [18] suggested that although ethnic differences in SJT performance exist, compared to other selection tools, SJT mean score differences tended to be of a lower magnitude compared to those of cognitive tests. These findings suggest that, when compared to alternative selection methods, SJTs tend to show less adverse impact on candidates from minority ethnic groups. Of the literature available on the use of SJTs in healthcare however, the vast majority of research is based on Western samples and very little research has been conducted in exploring potential scores differences regarding ethnicity in countries with a very different ethnic diversity, such as in South East Asia.

## The Indonesian context for selection

Similar to many countries, the traditional approach to medical student selection in Indonesia has relied heavily on prior academic attainment. The methods to select medical students in Indonesia are predominantly comprised of cognitive ability tests, such as aptitude tests, or mathematics and natural sciences subject tests [19]. In terms of policy, however, the Indonesian Medical Education Act Number 20 Year 2013 clearly states that the medical student admissions process should consider not only a candidate’s academic achievements, but also their non-academic attributes. Like many other countries, the issue of professionalism, is frequently identified as a major reason behind patient complaints and malpractice lawsuits [20]. When tracing the career path of these identified doctors, the origin of their unprofessional behaviour often can be tracked back to incidents relating to poor professionalism when they were in medical school. Papadakis et al. [21] proposed that those demonstrating unprofessional behaviour as students have a strong tendency to exhibit unprofessional behaviour in subsequent clinical practice. Therefore, there is a growing emphasis on assessing important non-academic attributes in medical school admissions. Despite this increasing recognition of the importance of selecting medical students based on their non-academic attributes, to date, there are no published studies exploring selection based on non-academic attributes in Indonesia. One of the underlying reasons for the limited use of non-academic tests in this context relate to concerns regarding potential adverse impact of such tests on candidate performance, given the diverse demographic profile in the country.

Indonesia is an archipelago country consisting of more than 300 ethnic groups with Javanese as the main ethnic group (41% of total population). There are five main islands and the most populated (more than 50% of total population) is Java, which has a well-developed education sector (including primary schooling and higher education). The existence of many different ethnic groups and apparent inequality in terms of income and education pose significant challenges in dealing with fairness and widening participation in medical school selection. Consequently, in Indonesia, with such diverse socio-cultural demographics, it is critical that selection methods that do not favour certain groups are used.

In this study, we aim to explore the development and piloting of an SJT to assess non-academic attributes for medical student selection at the Faculty of Medicine, Universitas Indonesia (FMUI). The FMUI is a state funded university in which the main selection methods assess academic attributes and attainment. In addition, we aimed to evaluate whether the piloting of an SJT would also confer diversity benefits regarding gender, socio-economic status and ethnicity, potentially replicating the emergent findings from a Western context.

## Method

### Development of the SJT

In line with good practice SJT design [9, 22, 23], the development of an SJT for use in the FMUI started by defining the test specification, which outlines key aspects such as the non-academic domains to be assessed, the test length and the context within which scenarios are set. Based on an analysis of the undergraduate curriculum, three domains were included in the test specification: professionalism, effective communication and self-awareness. The professionalism domain relates to the competency to behave according to moral values and integrity, and to protect the vulnerable. The self-awareness domain includes acting within one’s own limitations, responding to feedback, and a willingness to continuously learn. Effective communication is the ability to communicate with empathy, compassion and politeness, and sensitivity towards the diverse needs and background of others. The undergraduate curriculum has seven main competency domains based on the national standard of competency; the three domains above are related to the non-academic domains set out, while the rest of the competency domains are academic, and assessed through other selection methods. Thirty faculty members were recruited to write scenarios and response items for the pilot SJT. Each faculty member were either involved in coordinating educational courses; members of the student counselling team; or those involved in teaching empathy, communication and professionalism. The writing process was facilitated by researchers experienced in SJT development, including three of the authors (DS, AF, SY).

A total of 112 initial SJT scenarios were developed, all of which were set in a healthcare/learning context, answerable by high school leaver candidates and designed to assess one of the target attributes as defined in the test specification [24, 25]. An example of an SJT scenario is provided in Table 1. Each SJT scenario consists of a short paragraph (approximately 50–100 words to reduce cognitive load) that describes a situation likely to be encountered by students, followed by between five and eight different responses depending on the scenario presented (consisting of between 5 and 10 words each). Test takers are asked to rate each of these responses individually in terms of appropriateness, with one being very appropriate through to four being very inappropriate, which follows a previously validated methodology [9]. This knowledge-based instruction format SJT was chosen because it was less susceptible to faking and more appropriate in medicine due to its lower susceptibility to coaching effects [9]. In the design process, careful attention was paid to ensure the scenarios and responses were free from any cultural, ethnicity and gender biases. Once scenarios and responses had been developed, cross-writer evaluation was conducted to make any improvements. Content was then reviewed by the research team in terms of format and grammatical correctness.

**Table 1:** Example of an SJT scenario (translated from the original Indonesian language version)

Scenario	
Mario is a first-year medical student from a remote island in the east of Indonesia. He lives in the dormitory and has no family in Jakarta. Budi, his classmate, notices that Mario is very quiet and rarely participates in group discussion. When asked why he never participates in the discussion, Mario said that he couldn’t study because he doesn’t have a laptop.	
How appropriate is each of Budi’s responses below? (1 = very appropriate; 2 = appropriate but not ideal; 3 = inappropriate but not awful; 4 very inappropriate)	
Responses	
Q1. Report Mario’s problem to the teacher	
Q2. Lend Mario an unused laptop from his house	
Q3. Invite Mario to study together in his house	
Q4. Save some of his allowance to help Mario buy a laptop	
Q5. Tell other group members to not give tasks to Mario	
Q6. Let Mario find a solution by himself	

### Concordance stage

Consistent with previous research [9, 22, 23], a concordance study was conducted to establish the degree of agreement from subject matter experts to define an initial scoring key. A total of 112 scenarios (each including 5–8 nested items) were reviewed by 102 experts (including teaching staff, hospital staff, alumni and junior doctors) and 20 medical student representatives. Respondents were asked to comment on the relevance of each scenario, and to answer each question as though they were an applicant, to establish the agreed answer key. Psychometric experts analysed the data by using consensus analysis. This was measured by calculating the distribution of ratings provided by the panel. A threshold was set, whereby only items that had a minimum of 70% agreement on the same side of the rating scale (1–2, appropriate “side”; 3–4, not appropriate “side”), were deemed to have the necessary level of concordance to be progressed to the piloting phase. For most items, a consensus was reached on the expert scoring key (*N* = 412), however for the remaining 176 items, agreement was not gained on the correct answer. These 176 items were removed from the test.

### Initial psychometric evaluation with medical students

After eliminating the items that did not reach concordance (*N* = 176), as well as 44 scenarios which had fewer than 5 response items (*N* = 161), a total of 68 scenarios (374 items) were available to be piloted [9, 22, 23] with 436 first- and second-year medical students. Item responses were scored on a graded scale of 0 to 4 according to how ‘close’ the respondent’s response was to the expert scoring key. Data provided by 431 students were eligible for analysis, due to five students having a high amount of missing data. Ninety-two items were rejected following this analysis due to a poor correlation with overall test performance. Any scenarios which only contained one item (following removals) were also removed. Following this process, 282 items nested within 61 scenarios remained, with a Cronbach’s alpha value of .90. These items were spread across three domains: professionalism (105, 37%), effective communication (111, 39%) and self-awareness (66, 23%).

### Pilot testing of the SJT with applicants

**Fig. 1:**
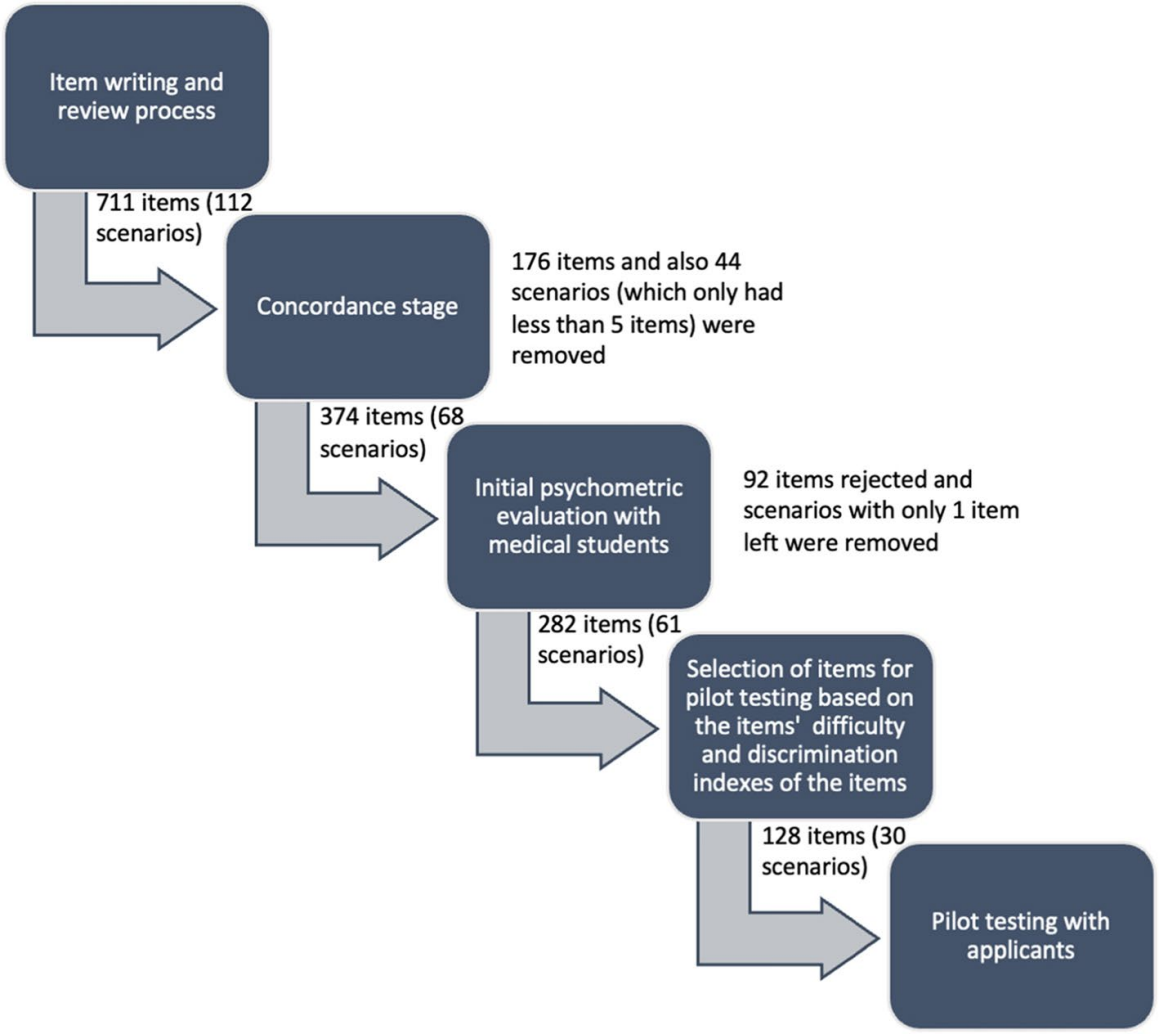
Summary of the SJT development and piloting process

Based on the level of difficulty (< .95) and discrimination (> .10) of each item, as obtained through the initial psychometric evaluation, 128 items across 30 scenarios were selected for pilot testing [9, 22, 23] among two cohorts of candidates applying to the International Class Programme during the years 2017–2018. The International Class Programme in FMUI is a special track in which students undergo one-year overseas study in partner universities to obtain the degree of bachelor of medical sciences or masters in research. Students completed the paper-based SJT for 1 h under invigilated exam conditions to ensure test security. They were also asked to provide their demographic details including gender, high school origin, parental educational background, and ethnicity. Students were informed of the nature of the test was a pilot study, and the results would not impact assessment decisions. Students were able to leave questions blank if they did not know the answer. The SJT development and piloting process is summarised in Fig. 1.

The items were presented under three domains: effective communication (62 items), professionalism (48 items) and self-awareness (18 items). To score the SJT, if a candidate gave a rating which matched the answer key, 4 points were obtained. The further the candidate rating from the answer key, the fewer points were received. In line with previous research [22], each candidate received a total SJT score. The maximum possible score was 512.

Using the total SJT score, independent t-tests were conducted to analyse differences between groups of candidates based on gender, whereas ANOVA tests were used for other grouping variables, i.e. socio-economic status (high school origin and parental educational background (elementary-high school, vocational-bachelor and masters-doctoral) and ethnicity (Indonesia, Javanese, Sumatran, Chinese, mixed ethnicity and other).

## Results

The validity of the test was established through a series of development and piloting stages. Content validity was confirmed through the development of test specification, item writing and reviews, and the concordance stage. Reliability analyses were conducted to examine internal consistency and SJT scores between demographic sub-groups were analysed to explore diversity issues.

Data was gathered from 446 and 508 candidates in 2017 and 2018 respectively. Table 2 shows the proportion of candidates, broken down by gender, socio-economic status (high school origin and parental educational background) and ethnicity.

**Table 2:** Candidate characteristics

Variable	Year 2017		Year 2018	
	n	%	n	%
**Gender**				
Male	304	68.16	347	68.31
Female	142	31.84	161	31.69
**High school origin**				
Java Bali	389	87.22	452	88.98
Sumatera	34	7.62	30	5.91
Kalimantan–Sulawesi	13	2.91	12	2.36
Overseas	10	2.24	14	2.76
**Father educational background**				
Elementary school – high school	28	6.28	33	6.50
Vocational - Bachelor	206	46.19	230	45.28
Masters - Doctoral	212	47.53	245	48.23
**Mother educational background**				
Elementary school – high school	47	10.54	54	10.63
Vocational - Bachelor	265	59.42	296	58.27
Masters - Doctoral	134	30.04	158	31.10
**Ethnicity**				
Indonesian	169	37.89	197	38.78
Javanese	130	29.15	136	26.77
Sumatran	52	11.66	59	11.61
Chinese	35	7.85	39	7.68
Other	33	7.40	42	8.27
Mixed ethnicity	27	6.05	35	6.89

The internal consistency (reliability) of the SJT was considered good for each of the two pilot years, with Cronbach’s alpha values of 0.80 and 0.81, respectively. The mean SJT scores were similar across both years with 419.9 (SD = 25.9) in 2017 and 422.6 (SD = 26.5) in 2018. The scores were then compared based on the diversity grouping variables, namely gender, socio-economic status and ethnicity (see Table 3). Equal variances can be assumed between groups given the non-significant results of the homogeneity of variance tests.

**Table 3:** SJT score differences for gender, socio-economic status (high school origin and parental background) and ethnicity

Variable	n	Year 2017			n	Year 2018		
		Mean score	SD	Mean score comparison		Mean score	SD	Mean score comparison
**Gender**								
Male	142	411.89	27.14	Independent t-test, *t*(444) = 4.561, *p* = 0.000*, *d* = 0.455	161	414.46	24.77	Independent t-test, *t*(506) = 4.78, *p* = 0.000*, *d* = 0.462
Female	304	423.62	24.40		347	426.31	26.54	
**High school origin**								
Java Bali	389	420.68	25.46	ANOVA test, *F*(3,442) = 1.582, *p* = 0.193, η^2^ = 0.011	452	422.48	26.90	ANOVA test, *F*(3,504) = 0.318, *p* = 0.812, η^2^ = 0.002
Sumatera	34	411.44	30.67		30	425.83	25.98	
Kalimantan–Sulawesi	13	414.92	24.29		12	423.00	23.67	
Overseas	10	424.00	22.85		14	417.57	18.53	
**Father educational background**								
Elementary school – high school	28	413.96	35.01	ANOVA test, *F*(2,443) = 0.789, *p* = 0.455, η^2^ = 0.004	33	416.21	21.71	ANOVA test, *F*(2,505) = 1.534, *p* = 0.217, η^2^ = 0.006
Vocational - Bachelor	206	420.43	24.86		230	421.70	26.50	
Masters - Doctoral	212	420.14	25.46		245	424.20	27.10	
**Mother educational background**								
Elementary school – high school	47	415.04	27.44	ANOVA test, *F*(2,443) = 1, *p* = 0.369, η^2^ = 0.005	54	415.20	25.16	ANOVA test, *F*(2,505) = 2.333, *p* = 0.098, η^2^ = 0.009
Vocational - Bachelor	265	420.09	26.22		296	423.33	27.26	
Masters - Doctoral	134	421.18	24.53		158	423.61	25.39	
**Ethnicity**								
Indonesian	169	423.17	24.55	ANOVA test, *F*(5,440) = 2.147, *p* = 0.059, η^2^ = 0.024	197	423.79	24.56	ANOVA test, *F*(5,502) = 1.321, *p* = 0.254, η^2^ = 0.013
Javanese	130	417.80	23.38		136	423.15	27.26	
Sumatran	52	411.58	28.35		59	416.97	29.89	
Chinese	35	423.43	30.70		39	425.72	26.00	
Other	33	417.27	28.01		42	416.50	29.96	
Mixed ethnicity	27	423.93	28.13		35	426.40	24.09	

Levene’s tests indicated equal variances (*p* value ranging from 0.60 to 0.899) which rendered the ANOVA results meaningful although there were large sample size differences among groups

*Denotes significant differences/correlations among variables (*p* < .05)

Regarding gender, significant differences were found between male and female candidates in 2017 and 2018 (*p* = 0.000) with females performing better than males. There were no significant differences between SJT scores for socio-economic status (high school origin and parental educational background) and ethnicity.

## Discussion

This exploratory study involved the design of an SJT (using a previously validated methodology) as part of selection into an undergraduate medical programme in Indonesia. The aim of this study was to explore the potential impact of an SJT on diversity issues, relating to gender, ethnicity and socio-economic status, which are important considerations in medical selection. Previous research on this topic has arisen mainly from north America, Europe and Australasia. To the author’s knowledge this is the first study regarding the impact of SJTs on diversity in a South East Asian context, which has a significantly different demographic profile of medical school applicants.

Our findings, of course, must be interpreted with caution given the uneven distribution of each variable group. The disproportionate distribution of each variable group reflects the actual situation in which applicants in each category are frequently dominated by certain group of candidates with a certain background. However, our pilot study offers some important new insights in SJT research. The study findings replicate previous research, in that females tend to perform marginally better on SJTs than males, and that there were no differences in SJT performance between different socio-economic groups (based on high school origin and parental educational background). However, unlike previous studies, our results do not find significant differences on SJT performance for different ethnic groups. Each diversity aspect is discussed in turn.

### Gender

As expected, our study revealed differences in SJT scores based on gender, whereby female candidates performed better on the SJT when compared to their male counterparts. This finding is consistent with other research in different geopolitical contexts [12, 13, 26]. Recent findings have also demonstrated that using an SJT, as part of screening for medical student selection in North America, were beneficial in enabling a higher proportion of female candidates, who have typically been underrepresented in medicine in the past, to progress to the next selection phase [6]. However, it is important to note that there now are increasing numbers of female medical students in many countries, so this is a consideration when using an SJT in how scores are weighted and combined with other selection methods in practice. One possible explanation for why females tend to outperform males on SJTs is that SJTs measure (to some extent) aspects of personality where females typically report greater conscientiousness, agreeableness and sensitivity when compared to males [26, 27]. Similarly, in Weekley and Jones’s study [28] where females tended to perform better on an SJT, they argued that this was due to many SJT items in their study relating to interpersonal conflicts or interactions. A more recent study [29] examined the use of an SJT in teacher selection, comparing both a text-based and a video-based SJT. When considering the text-based SJT, the findings were similar to those observed here; it was demonstrated that the differences between females and males were found in the items which related to conscientiousness and emotion regulation. In contrast, when considering performance on the video-based SJT, Bardach et al. [30] identified no statistically significant differences in performance when comparing males and females. As the authors note, this suggests that text-based and video-based SJT scenarios may be interpreted and responded to in different ways [31]. Further research is clearly warranted to understand the diversity impact regarding gender relating to the modality of administration for an SJT.

### Socio-economic status (SES)

Our results show no differences in SJT scores based on high school origin, suggesting that the current format may support widening diversity in medical school enrolment from all regions of Indonesia. This finding is also confirmed by the fact that parental educational background does not affect performance on the SJT in this study. Juster et al. [6] studied an online video-based SJT of non-academic attributes, which demonstrated smaller sub-group differences regarding socio-economic status in scores compared to cognitively-oriented tests, where those from a higher SES tended to perform better in both types of test, but significantly more so on the cognitive test. Bardach et al. [29] showed that teacher applicants from lower SES did not obtain lower scores on their SJT compared than those of the higher SES. Weekley and Jones [28] argued that SJTs assess tacit knowledge which is considered to be independent of cognitive ability and developed through socialisation experiences. As such, the nature and specific design parameters of an SJT, will contribute to its ability to have more or less adverse impact regarding socio-economic background, and thus demonstrate the potential use of SJTs to narrow, or even close, the gap between those from different SES groups. This is important, since widening participation is one of the key issues in medical school selection identified in the review of selection system of seven Asian countries [19] as one of the means to increase the diversity of the medical student intake and the doctor population. Research shows that the use of cognitive ability tests benefits candidates with higher socio-economic status [2] which is likely related to their exposure to higher quality educational resources such as specific training interventions [4]. In addition, SJTs can be designed to be less susceptible to coaching effects due to their diverse content/domain and complexity of the response format [32] and the involvement of expert judgments in determining the key [33]. Therefore, the use of such non-academic tests, targeting important interpersonal attributes required to be a successful clinician, could support the objective of increasing the diversity of medical students.

### Ethnicity

A key consideration when choosing a selection method is fairness, including differential performance based on ethnicity. Patterson et al. [3] highlighted the potential benefit of SJTs to reduce subgroup differences compared to cognitively-oriented assessments. The current study has further supported this, in that there were no statistically significant differences in SJT scores based on ethnic subgroups. Previous studies have shown varied results; Weekley and Jones [28] identified slightly higher performance of minority ethnic groups on an SJT, whereas Bardach et al. [30] and Weekley et al. [34] demonstrated that those from majority ethnic groups tended to perform better on SJTs, when compared to minority ethnic groups. Juster et al. [4] found higher SJT scores were obtained by non-Hispanic and non-Latino respondents in a North American medical school selection context; though, the ethnic subgroup differences were significantly smaller when compared to those observed when using academic metrics. The underlying mechanism is still unclear, but is possibly related to the increased cognitive load, when compared to SJTs. The findings from Nguyen and McDaniel [35] strengthen this argument since larger subgroup differences are found when SJTs using knowledge based instructions (choosing the best and worst options) were compared to behavioural tendency SJTs (indicating what would the respondent most or least likely do). The knowledge-based instruction approach is less open to faking because it correlates more strongly with cognitive ability, whereas behavioural tendency instructions correlate more highly with personality traits and are thus more susceptible to faking [36]. The SJT used in the current study is a knowledge-based instruction (procedural knowledge) type SJT, thus it may be expected that this type of SJT would cause greater differences among ethnic subgroups. However, the results in this study have suggested otherwise. Notwithstanding the need to better understand the causal factors regarding adverse impact and SJTs, evidence suggests that the use of an SJT as part of selection has the potential to increase diversity in medical schools in this context, and ultimately, in the healthcare workforce.

Our pilot evaluation of an SJT in this study is demonstrated to not advantage any particular groups based on the high school origin, parental educational background, or ethnicity. Indonesia is an archipelago country consists of five major islands where Java is the most populous island comprising just over 50 % of Indonesia’s total population. The density of population in the island of Java is also a reflection of how Java is the most developed island, thus it can be hypothesized that candidates coming in from outside Java may be disadvantaged. However, our preliminary data of our locally designed SJT has demonstrated otherwise; there are no significant differences among the subgroups based on the high school region. The Gini index of Indonesia in 2019 is 38.2 according to data from the World Bank. Gini index, ranging from 0 to 100, is one of the indicators of the level of inequality in one country, and the lower the index the more equal the people are. The Indonesia’s Gini index shows that there is still prominent inequality in the country. Notwithstanding the fact that the current study involved only one population of medical school candidates, it is worth noting that the SJT has shown potential to be an acceptable admission test in a country as diverse as Indonesia.

### Limitations

The SJT under study was a pilot test in a single institution, thus we are not able to provide results regarding the broader practical contribution of SJTs in selection in this setting and for the Indonesian context more widely. In addition, it was not possible to conduct any comparative analysis with the results of other selection methods utilized in the institution, which would also inform the added value of using an SJT. Despite these limitations, the results provide an early indication that SJTs can be a reliable tool for selecting undergraduate medical school candidates in this geopolitical context.

### Further research

To the best of our knowledge, there are very few studies on SJTs in an Asian medical education context. Therefore, our study also contributes to the literature by examining the development of SJTs based on the understanding of what constitutes the most important non-academic attributes in each unique setting. Although the test specification was developed based on the FMUI’s undergraduate curriculum, they can also be used in other medical schools in Indonesia with contextual modification, since the current SJT specification is constructed based on the national standard for medical doctor competencies. Further studies should examine the application of SJTs in different contexts with a broader range of variables representing diversity, as well as its predictive validity over time, preferably using non-academic outcome indicators of performance in a workplace setting.

## Conclusions

This research, to design and pilot an SJT which assesses non-academic attributes, has demonstrated that an SJT has the potential to be a reliable selection tool for undergraduate medical student admissions in an Indonesian context. The thorough development process, involving input from subject matter experts and medical students, as well as applicants, has supported the underpinning content validity of the use of an SJT in this context. The resulting preliminary analysis has demonstrated findings similar to those reported elsewhere internationally, in terms of the minimal impact on demographic sub-groups; considering SES and ethnicity. Therefore, this research supports the emerging literature on the potential use of SJTs to support widening participation into medicine, in this unique geopolitical context.

## Data Availability

The anonymous datasets used and/or analyzed during the current study are available from the corresponding author upon reasonable request. The SJT items belongs to the institution and are not to be released.

## Abbreviations

SJT: Situational Judgment Test
UCAT: University Clinical Aptitude Test
SES: Socio-economic status
